# Supplementary material for: Evaluation of Outcomes following Reduction in Targeted Fluid Administration in Major Burns
Source: Eur Burn J. 2023 May 29;4(2):234–47. doi: 10.3390/ebj4020021 (PMC11571837; doi:10.3390/ebj4020021)
Supplement: Supplementary file 1 [file ebj-04-00021-s001.zip › ebj-2320864-supplementary.pdf]

Figure S1. Fluid distribution by weight.

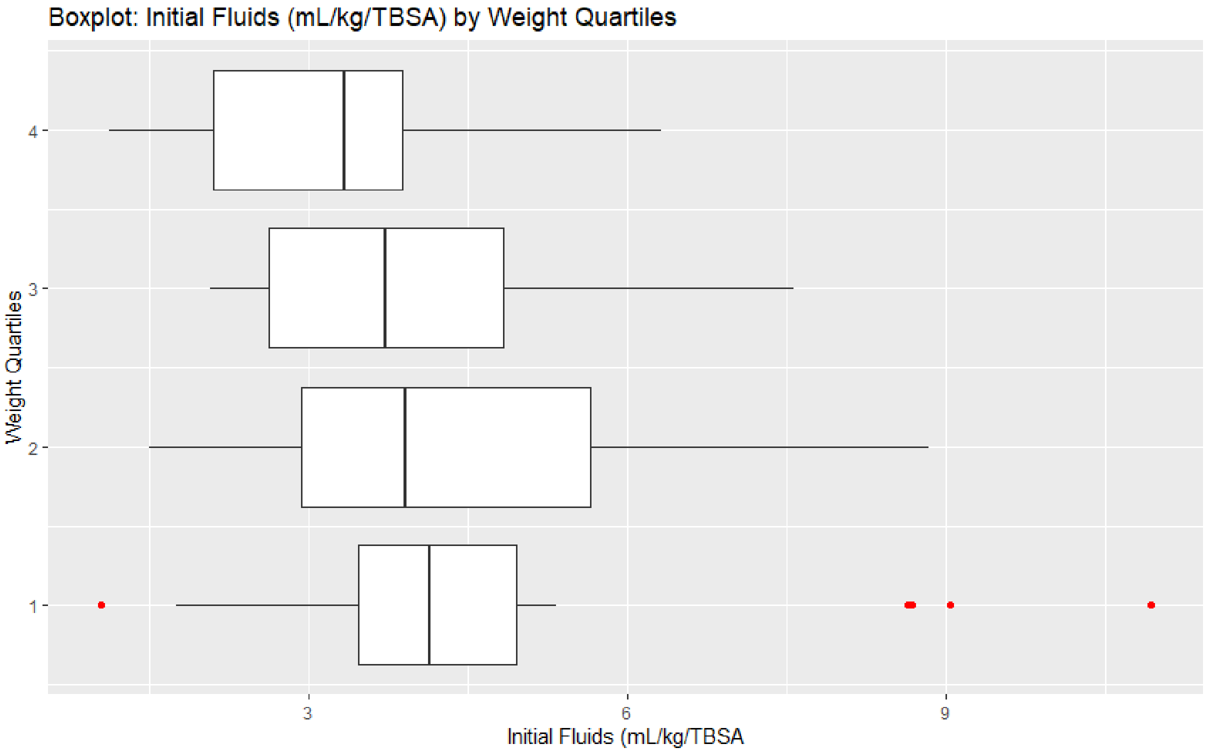

Table S1. Mortality Analysis Logistic Regression.

Feature	Odds Ratio	*p*-value
Age	1.11	**< 0.001**
TBSA	1.09	**< 0.001**
After change in fluid goals	1.76	0.44

Feature	Odds Ratio	*p*-value
Age	1.12	**< 0.001**
TBSA	1.09	**< 0.001**
After change in fluid goals	1.66	0.49
First 24-hour intake as mL/kg/TBSA	0.86	0.44

Table S2. Survival Analysis Cox Proportional Hazards.

**Feature**	**Odds Ratio**	***p*-value**
Age	1.09	**< 0.001**
TBSA	1.08	**< 0.001**
After change in fluid goals	1.21	0.72

**Feature**	**Odds Ratio**	***p*-value**
Age	1.1	**< 0.001**
TBSA	1.08	**< 0.001**
After change in fluid goals	1.17	0.77
First 24 hour intake as mL/kg/TBSA	0.83	0.22

Table S3. ARDS Development Logistic Regression.

**Feature**	**Odds Ratio**	***p*-value**
Age	1.03	0.1
TBSA	1.03	**0.03**
After change in fluid goals	2.56	0.12

**Feature**	**Odds Ratio**	***p*-value**
Age	1.02	0.18
TBSA	1.03	**0.02**
After change in fluid goals	2.81	0.1
First 24 hour intake as mL/kg/TBSA	1.18	0.28

AKI in First 7 Days Logistic Regression.

**Feature**	**Odds Ratio**	***p*-value**
Age	1.04	**< 0.01**
TBSA	1.05	**< 0.001**
After change in fluid goals	0.55	0.23

**Feature**	**Odds Ratio**	**p-value**
Age	1.04	**< 0.01**
TBSA	1.05	**< 0.01**
After change in fluid goals	0.54	0.1
First 24 hour intake as mL/kg/TBSA	0.94	0.28
